# 806 Foot burns in diabetic patients: A systematic review

**DOI:** 10.1093/jbcr/irac012.355

**Published:** 2022-03-23

**Authors:** Katherine J Choi, Christopher H Pham, Clifford C Sheckter, Zachary J Collier, Haig A Yenikomshian, Warren L Garner, Justin Gillenwater

**Affiliations:** University of California Los Angeles, Los Angeles, California; University of Southern California, Cypress, California; Stanford University, Stanford, California; Keck School of Medicine, University of Southern California, Los Angeles, California; USC/LAC+USC Medical Center, Los Angeles, California; USC, Los Angeles, California; USC/LAC+USC Medical Center, Los Angeles, California

## Abstract

**Introduction:**

Patients with diabetes are prone to foot injuries and burns. Managing burned feet in patients with diabetes can be difficult due to multiple concomitant patient comorbidities that delay wound healing. Burn surgeons tasked with treating these complex foot burns understand that these patients are at risk of developing significant complications, such as infections and non-healing diabetic foot ulcers, which may ultimately lead to amputation. Although there are many studies that examine the causes and outcomes of diabetic foot ulcers and their management, there is a lack of consensus on how to best manage lower extremity burns in patients with diabetes.

**Methods:**

A systematic review was performed according to PRISMA criteria and identified 18 articles addressing the management of burned feet in patients with diabetes. Means and standard deviations of scale variables and frequencies derived from nominal and ordinal variables abstracted from the literature were compared between each group. A meta-analysis was attempted but existing data was not of sufficient quality for meaningful analysis, and data aggregation was done where applicable.

**Results:**

The three-database search identified 726 articles, which yielded 18 full text articles that met inclusion criteria. mean age of patients was 54 years (SD 7), and 80% (n=203) were male. Most (90%, n=179) had type II diabetes, and 10% (n=20) had Type I diabetes. Mean duration of diabetic disease was 11 years (SD 4), and mean A1c at admission was 9% (SD 1). Peripheral neuropathy (64%, n=123) was the most common comorbidity. Median TBSA burned was 2% (IQR 2), and scald (56%, n=155) was the most common mechanism of injury. Full thickness burns (70%, n=102) were most common, followed by partial thickness (28%, n=41), and superficial thickness (2%, n=2). A majority (55%, n=88) had burn wound infection identified at admission. Patients had a median delay in presentation from time of injury to hospital admission of 4 days (IQR 4.5). Sixty percent of patients received surgical treatment via excision and grafting, had a median length-of-stay of 20.5 days despite a median total body surface area involvement of 2%, and experienced sequelae of impaired wound healing such as infections, graft loss, and need for further surgery. Ultimately, 23% of patients had significant wound healing issues that resulted in amputation.

**Conclusions:**

Excision and grafting may not be the optimal approach for managing foot burns in patients with diabetes, many of whom are at high risk of amputation. Non-operative management of foot burns in patients with diabetes should be explored as a method to decrease amputations in patients with foot burns.

